# Outcomes of total hip arthroplasty in obese patients with and without preoperative weight loss: A systematic review and meta‐analysis

**DOI:** 10.1002/jeo2.70651

**Published:** 2026-01-21

**Authors:** Nils Meissner, Sonia Ramos‐Pascual, Katharina Ortwig, Floris van Rooij, Daniel Schrednitzki, Johannes Stoeve, Andreas M. Halder

**Affiliations:** ^1^ Department of Orthopaedic Surgery Sana Hospital Sommerfeld Kremmen Germany; ^2^ ReSurg SA Nyon Switzerland; ^3^ German Society for Orthopedics and Trauma Surgery Berlin Germany; ^4^ Department of Orthopaedic, Trauma, Hand and Reconstructive Surgery Sana Klinikum Lichtenberg Berlin Germany; ^5^ Department of Orthopaedic and Trauma Surgery St. Marien‐ und St. Annastiftskrankenhaus Ludwigshafen Germany

**Keywords:** obese patients, systematic review, total hip arthroplasty, weight change, weight reduction

## Abstract

**Purpose:**

Obesity is often considered a relative contraindication to total hip arthroplasty (THA) due to presumed increased perioperative and postoperative risk. Consequently, obese patients are often advised to lose weight prior to THA. However, the effect of preoperative weight loss on THA outcomes remains uncertain. This meta‐analysis compared outcomes in obese patients who lost weight preoperatively with those who did not.

**Methods:**

This review was performed according to Preferred Reporting Items for Systematic Reviews and Meta‐analysis (PRISMA) guidelines and registered in PROSPERO. Medline and Embase were searched on 1 February 2025. Two reviewers independently screened and extracted data from studies comparing outcomes in obese patients undergoing primary THA with and without preoperative weight loss. Outcomes of interest (complications, infections, readmissions, reoperations and revisions) were pooled via Freeman–Tukey double arcsine transformations using inverse‐variance weighting within a random‐effects model framework to calculate estimates of proportions and their corresponding *p* value.

**Results:**

Of 2896 identified references, 8 studies were included, resulting in 4848 patients with preoperative weight loss and 78,860 patients without. Interventions included bariatric surgery (1 study), non‐surgical measures (2) and unspecified methods (5). There were no significant differences in outcomes between groups, with regards to complication rates in the short‐term (weight loss: 14% vs. control: 8%, *p* = 0.163) or mid‐term (5% vs. 8%, *p* = 0.568), prosthetic joint infection rates in the short‐term (5% vs. 4%, *p* = 0.458) or mid‐term (6% vs. 4%, *p* = 0.289), reoperation rates in the short‐term (2% vs. 1%, *p* = 0.840) or mid‐term (7% vs. 4%, *p* = 0.139), revision rates in the short‐term (1% vs. 1%, *p* = 0.401) or mid‐term (3% in both groups, *p* = 0.906) and readmission rates (5% vs. 4%, *p* = 0.077).

**Conclusions:**

Preoperative weight loss in obese patients undergoing THA does not reduce the risk of postoperative complications, infections, readmissions, reoperations or revisions compared with obese patients who did not lose weight preoperatively. These findings question routine weight loss requirements and underscore the need for individualized risk assessment over body mass index alone.

**Level of Evidence:**

Level IV.

**Abbreviations**
95% CI: 95% confidence intervals
BMI: body mass index
HHS: Harris Hip Score
iHOT12: International Hip Outcome Tool
MMAT: Mixed Methods Appraisal Tool
OHS: Oxford Hip Score
OR: odds ratio
PJI: prosthetic joint infection
PRISMA: Preferred Reporting Items for Systematic Reviews and Meta‐analysis
SSI: surgical site infection
THA: total hip arthroplasy
TJA: total joint arthroplasty
TJRR: Total Joint Replacement Registry
VAS: visual analogic scale

## INTRODUCTION

Primary total hip arthroplasty (THA) is a successful procedure that has demonstrated excellent mid‐ to long‐term outcomes [19]. However, obese patients scheduled for THA are often required to postpone surgery until they achieve weight reduction [53], as obesity is associated with an increased risk of complications, including deep infection, venous thromboembolism, hip dislocation, aseptic loosening and blood transfusions [9, 33, 50, 71]. This is particularly concerning given the rising prevalence of obesity [13], as this sub‐population may derive substantial benefit from THA.

Since obesity is a modifiable risk factor for patients undergoing THA [9], it is important to understand the available weight loss interventions and their impact on outcomes after primary THA. Several reviews have reported on specific weight loss interventions prior to THA, including bariatric surgery and non‐surgical weight loss [47, 51, 55, 69, 75]. However, most reviews included both THAs and total knee arthroplasties (TKAs), few reported outcomes after THA [42, 73, 86] and none pooled the data into a meta‐analysis to directly compare the effect of weight loss on outcomes regardless of the weight‐loss intervention.

As such, the purpose of the present meta‐analysis was to synthesize, critically appraise and systematically review published studies that compared outcomes of primary THA in overweight or obese patients who underwent preoperative weight loss versus those who maintained the same weight. It was hypothesized that overweight or obese patients who achieved weight reduction prior to THA would experience superior clinical outcomes and lower complication rates following THA compared to overweight or obese patients who maintained the same weight.

## MATERIALS AND METHODS

The protocol for this meta‐analysis was submitted to PROSPERO prior to commencement of the study (registration number CRD42025645038) and follows the Preferred Reporting Items for Systematic Reviews and Meta‐analysis (PRISMA) guidelines [58]. A structured electronic literature search was conducted on 1 February 2025 using the Medline (PubMed) and Embase databases, applying the keywords presented in Appendix 1.

### Study selection

After removal of duplicate records, two researchers (N.M. and S.R.P.) each screened all titles and abstracts to assess eligibility based on predefined inclusion and exclusion criteria. Discrepancies regarding the suitability of records were resolved through review and consensus, or by involving a third researcher (K.O.). Full‐text review of studies meeting the initial screening criteria was conducted independently by the same two researchers, with disagreements again resolved by consensus or by involving the third researcher. To ensure comprehensive inclusion of relevant literature, two active high‐volume hip surgeons (A.M.H. and J.S.) were consulted to identify potentially eligible studies that have not been captured through the database searches. Additionally, the references of each included study were screened for further relevant and eligible articles.

Articles were included in the present meta‐analysis if they were prospective or retrospective clinical studies comparing outcomes following primary THA in overweight/obese (body mass index [BMI] > 25 kg/m^2^) patients, who underwent preoperative weight loss versus those who maintained their baseline weight, regardless of the weight loss intervention (pharmacological, dietary, surgical, exercise‐based, etc.). For patients in the weight loss group, we included both patients who were overweight/obese at the time of THA, as well as those who had lost weight and were no longer overweight/obese at the time of THA. Outcomes of interest were complications, readmissions, reoperations, revisions and all clinical outcomes.

Articles were excluded from the present meta‐analysis if they (i) reported on a weight loss intervention, but did not specifically state that patients lost weight, or did not provide pre‐intervention and post‐intervention BMI; (ii) reported on combined results for THA and TKA, where the outcomes were not presented separately for each surgery; (iii) were non‐comparative studies or case series. Although the PROSPERO submission stated that non‐comparative studies would also be included in this review, the authors decided to finally only include comparative studies, as there were sufficient articles available and this increased the level of evidence of the findings; (iv) were case reports, reviews, editorials, expert opinions, letters to the editor or conference proceedings; (v) reported on cadavers, animals, in vitro experiments or in silico simulations or (vi) were written in languages other than English or German, to avoid translation errors.

### Data extraction and quality assessment

Data extraction was performed independently by two researchers (L.N. and M.H.), and their results were compared. Any discrepancies in values between researchers were resolved through review and consensus, or through consultation of a third researcher (S.R.P.) when needed. The following data were extracted from the included studies: author(s), journal, year of publication, article type (e.g., prospective comparative study), ethical approval and conflicts of interest. Cohort characteristics were retrieved, including indication for surgery, number of patients, sex, age, weight and BMI. Type of weight loss intervention, definition of weight loss (e.g., >5%), follow‐up time, complications (including prosthetic joint infections [PJIs], surgical site infections [SSIs], sepsis, aseptic loosening and dislocations), readmissions, reoperations, revisions and clinical outcomes (e.g., Harris Hip Score [HHS], Oxford Hip Score [OHS], International Hip Outcome Tool [iHOT12], pain and satisfaction on visual analogic scale [VAS]) were retrieved.

Methodological quality of the eligible studies was independently assessed by two researchers (L.N. and M.H.) using the Mixed Methods Appraisal Tool (MMAT) [27, 28]. Any discrepancies in appraisal between researchers were resolved through review and consensus, or by involving a third researcher (S.R.P.). The methodological quality scores of each study were calculated by aggregating affirmative responses to the relevant MMAT criteria, which can be answered as ‘yes’, ‘no’ or ‘can't tell’. Studies with an MMAT score ≥6 ‘yeses’, with the first two questions rated as ‘yes’, were considered of high quality [8, 85].

### Statistical analysis

Categorical outcomes were reported as events and proportions, while continuous outcomes were reported as means, standard deviations and ranges. Data on infections, complications, readmissions and revisions were pooled and illustrated using forest plots. To avoid combining widely differing follow‐up times, where appropriate, forest plots were stratified by follow‐up duration. Studies reporting outcomes at an early follow‐up (<3 months) were considered ‘short‐term’, while longer term follow‐ups (>24 months) were considered ‘mid‐term’. The stratification was chosen pragmatically based on the available data rather than predefined literature standards. Pooled estimates of proportions, odds ratios (ORs) and their 95% confidence intervals (95% CIs) were calculated using the Freeman‐Tukey double arcsine transformation and inverse‐variance weighting within a frequentist random‐effects model framework. A random‐effects model was selected because it incorporates both within‐study variance and between‐study heterogeneity, providing more appropriate weighting when true effects may vary across studies. Between‐study variance was estimated using the DerSimonian–Laird method, while Hartung–Knapp adjustment was not applied. Heterogeneity was evaluated by visual inspection of forest plots and by calculating the *I* ^2^ statistic, which quantifies the proportion of total variability attributable to heterogeneity rather than sampling error and its associated *χ* ^2^ test [26]. Funnel plots were not performed because the meta‐analysis contained fewer than ten studies. With such small numbers, funnel plots and associated tests can have low power and produce misleading conclusions about publication bias. *p* values < 0.05 were considered statistically significant. Statistical analyses were performed using R version 4.5.0 (R Foundation for Statistical Computing) using the meta package.

## RESULTS

### Literature search

**Figure 1:**
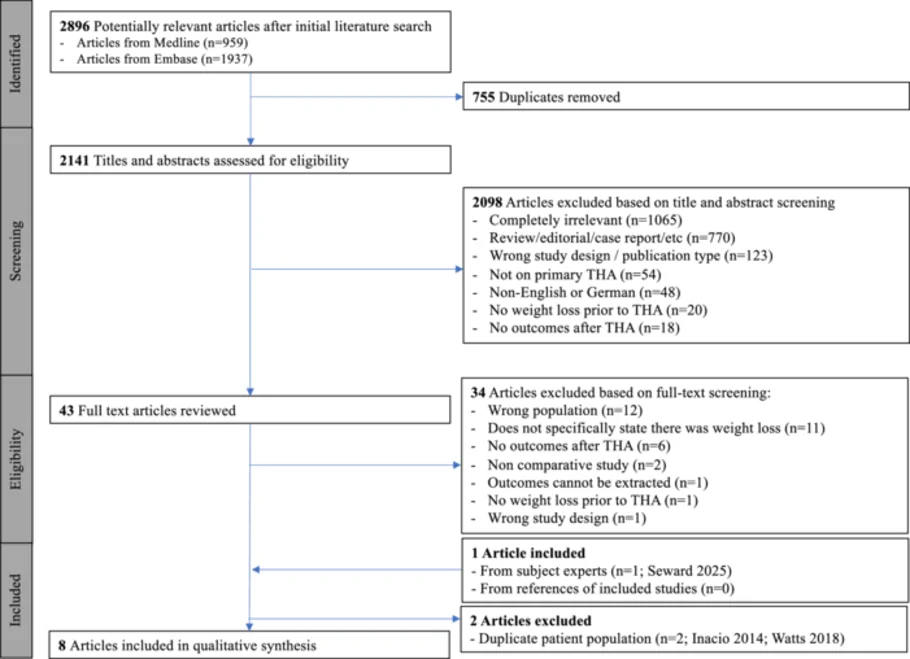
Flowchart presenting the electronic literature search and selected articles. THA, total hip arthroplasty.

The electronic literature search identified 2896 references, 755 of which were duplicates and subsequently removed (Figure 1). The remaining 2141 unique articles underwent title and abstract screening, and 2098 were excluded as they did not meet the inclusion criteria. The full text of the remaining 43 articles were screened, and a further 34 articles were excluded for the following reasons: 12 reported on the wrong population [24, 32, 37, 41, 43, 44, 46, 48, 56, 60, 61, 66], 11 did not specifically state that patients lost weight [7, 11, 34, 35, 36, 45, 49, 54, 57, 64, 80], 6 did not report outcomes after THA [2, 12, 18, 29, 38, 74], 2 were non‐comparative studies [59, 67], 1 did not present outcomes in an exploitable format [77], 1 did not report on weight loss prior to THA [21] and 1 employed an ineligible study design [68]. In addition, subject experts provided one further eligible study [70]. No further studies were identified through reference list screening. Two sets of studies were found to have considerable overlap in included patients. Inacio et al. [31] and Inacio et al. [30] both used the same large integrated healthcare Total Joint Replacement Registry (TJRR) database and had overlapping years of inclusion, while Seward et al. [70] and Watts et al. [81] both used the Mayo Clinic's institutional database and had overlapping years of inclusion. To avoid duplication, only the respective studies with the larger patient population were retained [30, 70]. As a result, eight articles were included in the present meta‐analysis [25, 30, 39, 52, 65, 70, 72, 84]. All were retrospective in design and published between 2014 and 2025 (Table 1). Six studies disclosed conflicts of interests, and one declared external funding (Appendix 2). Seven studies were conducted in the United States and one originated in France. Methodological quality, assessed using the MMAT, indicated that three studies scored 7 of 7 points, four scored 6 of 7 and one scored 5 of 7, thus seven of the eight included studies were of high methodological quality (Table 2).

**Table 1:** Characteristics of the included articles.

First author, year	Study type	Journal	Country	Database	Inclusion period for THA	Total number of patients	Indication for THA	BMI threshold used for inclusion	Weight loss intervention	Time period of weight loss	Funding	COI
Seward 2025	Retrospective	*JBJS Am*	USA	Institutional (Mayo Clinic)	2002–2019	2040	Primary OA	BMI ≥ 30 kg/m^2^	Bariatric surgery and others	Within 24 m of THA	Yes	Yes
Schmerler 2024	Retrospective	*J Arthroplasty*	USA	American College of Surgeons database	2013–2020	54,491	NR	BMI ≥ 35 kg/m^2^	NR	Within 6 m of THA	None	None
Shul 2024	Retrospective	*J Arthroplasty*	USA	PearlDiver database	2010–2020	15,753	NR	BMI 40–50 kg/m^2^	NR	Within 3–24 m of THA	NR	Yes
LaValva 2024	Retrospective	*J Arthroplasty*	USA	Institutional (Hospital for Special Surgery)	2016–2021	483	Primary OA	BMI ≥ 40 kg/m^2^	NR	NR	Yes	Yes
Middleton 2022	Retrospective	*J Arthroplasty*	USA	Institutional (Medical College of Wisconsin)	2015–2019	1589	OA and others	BMI < 40 kg/m^2^, >40 kg/m^2^	Non‐surgical	Within 24 m of THA	None	Yes
Wu 2022	Retrospective	*J Arthroplasty*	USA	Institutional (Duke University Medical Center)	2010–2020	337	OA	BMI ≥ 35 kg/m^2^	Non‐surgical	NR	None	Yes
Hernigou 2016	Retrospective	*CORR*	France	Institutional (Hospital Henri Mondor)	1990–2008	455	Primary OA, Dysplasia, Osteonecrosis, RA	BMI ≥ 30 kg/m^2^	Bariatric surgery	NR	NR	None
Inacio 2014	Retrospective	*J Arthroplasty*	USA	Total Joint Replacement Registry for a large integrated healthcare system	2008–2010	3808	OA	BMI ≥ 30 kg/m^2^	NR	NR	None	None

Abbreviations: BMI, body mass index; COI, conflict of interest; NR, not reported; OA, osteoarthritis; RA, rheumatoid arthritis; THA, total hip arthroplasty.

**Table 2:** Mixed Methods Appraisal Tool (MMAT) for quantitative non‐randomized studies.

First author, year	S1. Are there clear research questions?	S2. Do the collected data allow to address the research questions?	3.1. Are the participants representative of the target population?	3.2. Are measurements appropriate regarding both the outcome and intervention (or exposure)?	3.3. Are there complete outcome data?	3.4. Are the confounders accounted for in the design and analysis?	3.5 During the study period, is the intervention administered (or exposure occurred) as intended?	Total score
Seward 2025	Yes	Yes	No	Yes	Yes	Yes	Yes	6
Schmerler 2024	Yes	Yes	Yes	Yes	Yes	Yes	Yes	7
Shul 2024	Yes	Yes	Yes	Yes	Yes	Yes	Yes	7
LaValva 2024	Yes	Yes	No	Yes	Yes	Yes	Yes	6
Middleton 2022	Yes	Yes	No	Yes	Yes	Yes	Yes	6
Wu 2022	Yes	Yes	No	Yes	Yes	Yes	Yes	6
Hernigou 2016	Yes	Yes	No	Yes	Yes	No	Yes	5
Inacio 2014	Yes	Yes	Yes	Yes	Yes	Yes	Yes	7

### Weight loss interventions and patient characteristics

There were large variations across the eight included studies with regard to the BMI thresholds used for inclusion of patients. The BMI threshold was ≥30 kg/m^2^ in three studies, ≥35 kg/m^2^ in two studies, ≥40 kg/m^2^ in two studies and 40–50 kg/m^2^ in one study (Table 1). The type of weight loss intervention was bariatric surgery in one study and non‐surgical in two studies, while one study included mixed interventions (bariatric surgery and others), and four studies did not report on the type of weight loss intervention. The time period of weight loss varied across studies and ranged from within 3 months to within 5 years prior to THA.

In total, the eight studies reported on 78,860 obese patients, of whom 4848 patients had undergone weight loss prior to THA and 74,012 patients had not (Table 3). Individual cohort sizes ranged from 337 to 54,491 subjects per study.

**Table 3:** Preoperative patient characteristics of the included articles.

First author, year	Group explanation	Number of patients	Females	BMI before weight loss intervention	BMI at THA	Age at THA	Diabetes	Current tobacco user	Charlson comorbidity index	ASA 1	ASA 2	ASA 3	ASA 4
Seward 2025	No weight loss (within ±5 lb)	926	49%	35 ± 4	35 ± 4	66 ± 11		8.8%	5.1 ± 3.5	2.0%	56%	42%	0.0%
	Weight loss (5–10 pounds)	403	49%	36 ± 5	34 ± 5	67 ± 11		7.4%	5.4 ± 3.6	1.0%	57%	40%	1.0%
	Weight loss (10–20 pounds)	416	43%	37 ± 6	35 ± 6	68 ± 10		8.4%	5.7 ± 3.5	0.0%	50%	47%	2.0%
	Weight loss (≥20 pounds)	295	49%	40 ± 8	34 ± 7	66 ± 11		6.2%	5.9 ± 4.0	0.0%	43%	53%	4.0%
Schmerler 2024	No weight loss	54,388	55%		40 ± 4	62 ± 10	22%	12%		1.0%	34%	62%	3.0%
	Weight loss (BW > 10%)	103	57%		38 ± 5	64 ± 10	26%	15%		0.0%	30%	62%	8.0%
Shul 2024	No weight loss (BMI 40–50)	13,287	60%			61 (57–64)	59%	50%					
	Weight loss (BMI 45–50 reduced to <35 within 3 m of THA)	817	63%			63 (56–78)	61%	60%					
	Weight loss (BMI 45–50 reduced to <35 within 6 m of THA)	288	66%			63 (51–68)	64%	57%					
	Weight loss (BMI 45–50 reduced to <35 within 9 m of THA)	931	65%			63 (50–74)	62%	61%					
	Weight loss (BMI 45–50 reduced to <35 within 12 m of THA)	430	67%			62 (58–69)	64%	61%					
LaValva 2024	No weight loss (BW within ±5%)	336	60%		43 ± 3	60 ± 9		6.0%	0: 50% 1: 31% 2: 10% 3+: 9%	28%		72%	
	Weight loss (BW < 5%)	147	63%		40 ± 5	61 ± 9		5.0%	0: 63% 1: 26% 2: 6% 3+: 5%	49%		28%	
Middleton 2022	No weight loss (BMI < 40)	1387	58%	<40	29 ± 5	66 ± 11	15%		49.7 ± 27.9	53%		46%	0.6%
	No weight loss (BMI > 40)	96	55%	>40	44 ± 3	62 ± 10	37%		57.6 ± 22.4	16%		84%	0.0%
	Weight loss (BMI > 40 reduced to <40)	106	62%	>40	38 ± 2	62 ± 11	30%		55.5 ± 25.6	27%		72%	0.9%
Wu 2022	No weight loss (BMI within ±5%)	242	53%	<35: 12% 35–39.9: 64% 40–67: 24%	35–39.9: 79% 40–67: 21%	61 ± 12							
	Weight loss (BMI < 5%)	95	58%	35–39.9: 33% 40–67: 67%	35–39.9: 85% 40–67: 15%	60 ± 10							
Hernigou 2016	No weight loss, standard cup	215	54%		39 ± 5	72 ± 9							
	No weight loss, dual‐mobility or unconstrained cup	155	57%		40 ± 5	72 ± 13							
	Weight loss, standard cups	85	57%	42 ± 7	28 ± 4	71 ± 8							
Inacio 2014	No weight loss (BW within ±5%)	3076	54%		<30: 7% 30–35: 57% ≥35: 36%	<65: 50% ≥65: 50%	26%			52%		46%	
	Weight loss (BW ≤ 5%)	732	61%		<30: 33% 30–35: 43% ≥35: 24%	<65: 50% ≥65: 50%	27%			53%		45%	

Abbreviations: ASA, American Society of Anesthesiologists score; BMI, body mass index; BW, body weight; THA, total hip arthroplasty.

### Complications

**Figure 2:**
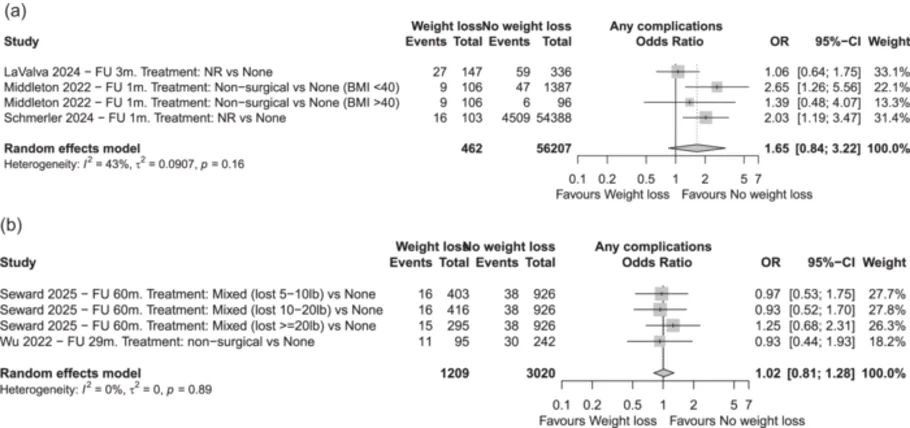
Forest plots presenting the overall complication rates in (a) the short‐term and (b) mid‐term. CI, confidence interval; FU, follow‐up; OR, odds ratio.

Overall complication rates were reported in five studies and ranged from 3.8%–18.4% in the weight loss group compared to 3.4%–17.6% in the no weight loss group (Table 4). There were no significant differences in the pooled proportion of short‐term overall complication rates between groups, although there was a trend for the weight loss group to have a higher complication rate (14% [95% CI = 9%–20%] vs. 8% [95% CI = 3%–15%], *p* = 0.163; OR = 1.65 [95% CI = 0.8–3.2], *I* ^2^ = 43%) (Figure 2a). Similarly, there were no significant differences in the pooled proportion of mid‐term overall complication rates between groups (5% [95% CI = 3%–8%] vs. 8% [95% CI = 1%–18%], *p* = 0.568; OR = 1.02 [95% CI = 0.8–1.3], *I* ^2^ = 0%) (Figure 2b).

**Table 4:** Outcomes reported in the included articles.

First author, year	Group explanation	Number of patients	FU (months)	Complications	Prosthetic joint infections	Surgical site infections	Aseptic loosening	Dislocations	Readmissions	Reoperations	Revisions
Seward 2025	No weight loss (within ±5 lb)	926	60 (24–228)	4.1%						4.3%	2.8%
	Weight loss (5–10 pounds)	403	60 (24–228)	4.0%						5.2%	3.0%
	Weight loss (10–20 pounds)	416	60 (24–228)	3.8%						5.5%	4.6%
	Weight loss (≥20 pounds)	295	60 (24–228)	5.1%						5.8%	3.4%
Schmerler 2024	No weight loss	54,388	1	8.3%		2.5%			4.3%	2.9%	
	Weight loss (BW >10%)	103	1	15.5%		2.0%			7.8%	5.8%	
Shul 2024	No weight loss (BMI 40–50)	13,287	3, 24		3 m FU = 4.0% 24 m FU = 4.9%	3 m FU = 4.2% 24 m FU = 5.6%	3 m FU = 0.5% 24 m FU = 1.1%				3 m FU = 0.9% 24 m FU = 1.4%
	Weight loss (BMI 45–50 reduced to <35 within 3 m of THA)	817	3, 24		3 m FU = 3.5% 24 m FU = 4.5%	3 m FU = 2.9% 24 m FU = 4.4%	3 m FU = 0.6% 24 m FU = 1.7%				3 m FU = 0.9% 24 m FU = 2.0%
	Weight loss (BMI 45–50 reduced to <35 within 6 m of THA)	288	3, 24		3 m FU = 7.3% 24 m FU = 8.3%	3 m FU = 5.6% 24 m FU = 7.6%	3 m FU = 1.7% 24 m FU = 2.4%				3 m FU = 1.0% 24 m FU = 1.7%
	Weight loss (BMI 45–50 reduced to <35 within 9 m of THA)	931	3, 24		3 m FU = 3.0% 24 m FU = 3.8%	3 m FU = 2.6% 24 m FU = 4.4%	3 m FU = 0.5% 24 m FU = 1.6%				3 m FU = 1.1% 24 m FU = 2.1%
	Weight loss (BMI 45–50 reduced to <35 within 12 m of THA)	430	3, 24		3 m FU = 5.8% 24 m FU = 7.0%	3 m FU = 4.7% 24 m FU = 6.3%	3 m FU = 1.4% 24 m FU = 1.6%				3 m FU = 1.2% 24 m FU = 1.9%
LaValva 2024	No weight loss (BW within ±5%)	336	3	17.6%	0.3%^a^			0.3%	3.9%	0.3%	0.3%
	Weight loss (BW < 5%)	147	3	18.4%	0%^a^			0.7%	4.8%	0.0%	0.0%
Middleton 2022	No weight loss (BMI < 40)	1387	0.2, 1, 3	3.4%	7 d FU = 0.3%^a^	3 m FU = 0.4%			1 m FU = 3%		
	No weight loss (BMI > 40)	96	0.2, 1, 3	6.3%	7 d FU = 0%^a^	3 m FU = 4.2%			1 m FU = 4.2%		
	Weight loss (BMI > 40 reduced to <40)	106	0.2, 1, 3	8.5%	7 d FU = 0%^a^	3 m FU = 2.8%			1 m FU = 7.5%		
Wu 2022	No weight loss (BMI within ±5%)	242	29	12.4%	2.9%				6.2%		5.4%
	Weight loss (BMI < 5%)	95	29	11.6%	6.3%				9.5%		5.3%
Hernigou 2016	No weight loss, standard cup	215	216 (180–228)					1 y FU = 6% 7 y FU = 9% 15 y FU = 13%		6.0%	
	No weight loss, dual‐mobility or unconstrained cup	155	144 (7–15)					1 y FU = 2% 7 y FU = 2% 11 y FU = 3%		1.3%	
	Weight loss, standard cups	85	132 (7–15)					1 y FU = 13% 7 y FU = 14% 11 y FU = 15%		15.3%	
Inacio 2014	No weight loss (BW within ±5%)	3076	3			1.5%			4.6%		
	Weight loss (BW ≤ 5%)	732	3			1.9%			4.8%		

Abbreviations: BMI, body mass index; BW, body weight; FU, follow‐up; THA, total hip arthroplasty.

^a^ Sepsis.

**Figure 3:**
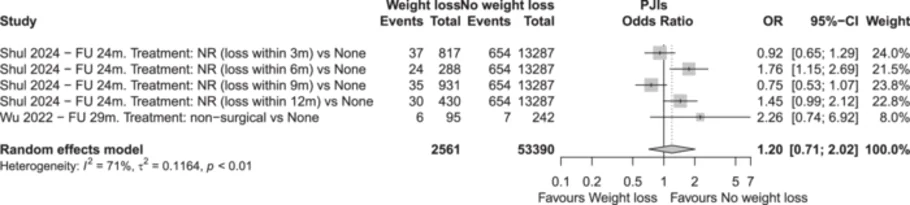
Forest plots presenting the PJI rates in the mid‐term. CI, confidence interval; FU, follow‐up; OR, odds ratio; PJIs, prosthetic joint infections.

PJI rates were reported in two studies and ranged from 3.0%–8.3% in the weight loss group compared to 2.9%–4.9% in the no weight loss group (Table 4). The pooled proportion of short‐term PJI rates was not calculated since only one study reported this variable. There were no significant differences in the pooled proportion of mid‐term PJI rates between groups (6% [95% CI = 4%–8%] vs. 4% [95% CI = 3%–6%], *p* = 0.289; OR = 1.2 [95% CI = 0.7–2.0], *I* ^2^ = 71%) (Figure 3).

**Figure 4:**
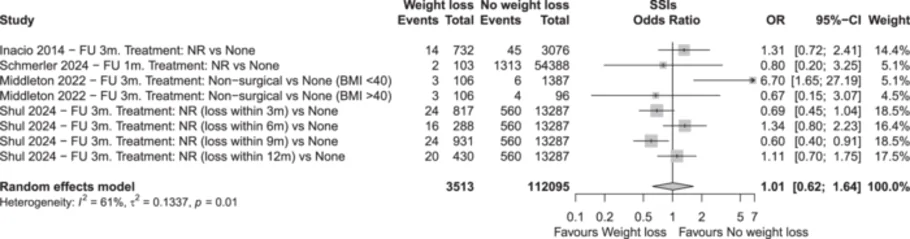
Forest plots presenting the SSI rates in the short‐term. CI, confidence interval; FU, follow‐up; OR, odds ratio; SSIs, surgical site infections.

SSI rates were reported in four studies and ranged from 1.9%–7.6% in the weight loss group compared to 0.4%–5.6% in the no weight loss group (Table 4). There were no significant differences in the pooled proportion of short‐term SSI rates between groups (3% [95% CI = 2%–4%] vs. 2% [95% CI = 1%–4%], *p* = 0.297; OR = 1.01 [95% CI = 0.6%–1.6%], *I* ^2^ = 61%) (Figure 4). The pooled proportion of mid‐term SSI rates was not calculated since only one study reported this variable.

### Readmissions

**Figure 5:**
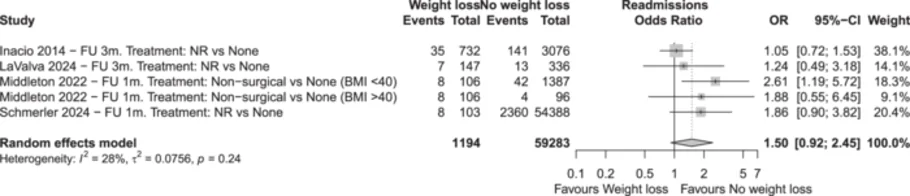
Forest plots presenting the readmission rates in the short‐term. BMI, body mass index; CI, confidence interval; FU, follow‐up; OR, odds ratio; NR, not reported.

Readmission rates were reported in five studies and ranged from 4.8%–9.5% in the weight loss group compared to 3.0%–6.2% in the no weight loss group (Table 4). There were no significant differences in the pooled proportion of short‐term readmission rates between groups (5% [95% CI = 4%–7%] vs. 4% [95% CI = 3%–5%], *p* = 0.077; OR = 1.50 [95% CI = 0.9–2.5], *I* ^2^ = 28%) (Figure 5). The pooled proportion of mid‐term readmission rates was not calculated since only one study reported this variable.

### Reoperations

**Figure 6:**
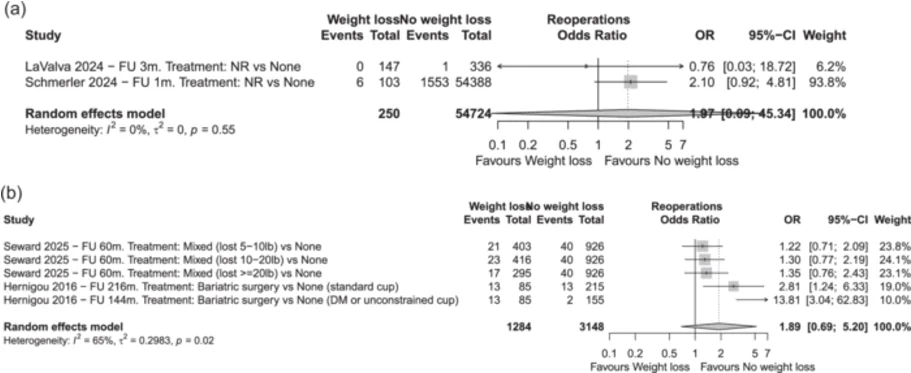
Forest plots presenting the reoperation rates in (a) the short‐term and (b) mid‐term. CI, confidence interval; DM, dual‐mobility; FU, follow‐up; OR, odds ratio; NR, not reported.

Reoperation rates were reported in four studies and ranged from 0.0%–15.3% in the weight loss group compared to 0.3%–6.0% in the no weight loss group (Table 4). There were no significant differences in the pooled proportion of short‐term reoperation rates between groups (2% [95% CI = 0%–12%] vs. 1% [95% CI = 0%–5%], *p* = 0.840; OR = 1.97 [95% CI = 0.1–45.3], *I* ^2^ = 0%) (Figure 6a). Similarly, there were no significant differences in the pooled proportion of mid‐term reoperation rates between groups, although there was a trend for the weight loss group to have a higher reoperation rate (7% [95% CI = 4%–10%] vs. 4% [95% CI = 2%–7%], *p* = 0.139; OR = 1.89 [95% CI = 0.7–5.2], *I* ^2^ = 65%) (Figure 6b).

### Revisions

**Figure 7:**
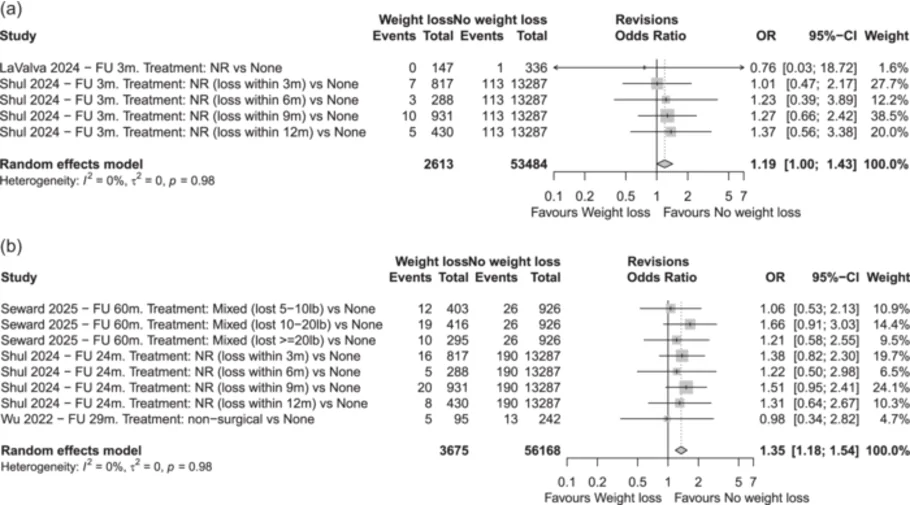
Forest plots presenting the revision rates in (a) the short‐term and (b) mid‐term. CI, confidence interval; FU, follow‐up; OR, odds ratio; NR, not reported.

Revision rates were reported in four studies and ranged from 0.0%–5.3% in the weight loss group compared to 0.3%–5.4% in the no weight loss group (Table 4). There were no significant differences in the pooled proportion of short‐term revision rates between groups (1% [95% CI = 1%–1%] vs. 1% [95% CI = 1%–1%], *p* = 0.401; OR = 1.19 [95% CI = 1.0–1.4], *I* ^2^ = 0%) (Figure 7a). Similarly, there were no significant differences in the pooled proportion of mid‐term revision rates between groups (3% [95% CI = 2%–3%] vs. 3% [95% CI = 1%–5%], *p* = 0.906; OR = 1.35 [95% CI = 1.2–1.5], *I* ^2^ = 0%) (Figure 7b).

### Clinical outcomes

None of the included articles reported on post‐THA clinical outcomes, such as HHS, OHS, iHOT12, pain or satisfaction with surgery.

### Effect of weight loss amount on outcomes

One of the included studies [70] reported outcomes stratified by the amount of weight loss (5–10, 10–20 and ≥20 lb) and found no association between the amount of weight loss and complication rates or revision rates, although reoperation rates tended to increase as weight loss increased (Table 4). Reoperation rates were 4.3% for the no weight loss group, compared to 5.2% for the weight loss group of 5–10 lb, 5.5% for the weight loss group of 10–20 lb and 5.8% for the weight loss group of ≥20 lb.

### Effect of timepoint at which weight loss occurred on outcomes

One of the included studies [72] reported outcomes stratified by the weight loss timepoint before THA (weight loss within 3 months from THA, 6, 9 and 12 months) and found no clear association between the timepoint at which weight loss occurred and crude rates of infection or revision (Table 4). However, multivariate logistic regression analyses indicated that patients who had weight loss 3, 6, and 9 months before surgery had significantly higher odds of developing PJI (OR: 2.15, 2.34, and 5.22, respectively; *p* < 0.001).

## DISCUSSION

The present meta‐analysis included eight retrospective studies that compared outcomes of primary THAs in overweight or obese patients who underwent preoperative weight loss versus those who maintained their baseline weight. The available data revealed that there were no significant differences in outcomes between groups, in terms of pooled proportions of complication rates, PJI rates, SSI rates, readmission rates, reoperation rates, nor revision rates, neither at the short‐ nor mid‐term. The hypothesis that overweight or obese patients who lost weight preoperatively would achieve superior outcomes after THA compared to those who did not was not confirmed by the available evidence. However, it is worth noting that most included studies did not control for important study‐level confounders, such as American Society of Anaesthesiologists (ASA) physical status, diabetes, or smoking status.

Several interventions are available for weight loss in overweight and obese patients, including both surgical and non‐surgical [1, 4, 5, 15, 17, 20, 23, 78]. Surgical interventions, such as gastric bypass (Roux‐en‐Y), sleeve gastrectomy, adjustable gastric banding and biliopancreatic diversion, have shown substantial and sustained weight loss [1, 5]. However, they are associated with inherent risks and require significant long‐term lifestyle modifications [1, 5]. In contrast, non‐surgical interventions, including pharmacotherapy, dietary interventions, behavioural therapy and physical activity, are generally safer but often result in less durable outcomes [4, 15, 17, 20, 23, 78]. Glucagon‐like peptide‐1 receptor agonists (GLP‐1) are modern pharmacotherapies that are gaining momentum for weight loss. A recent meta‐analysis of 47 randomized controlled trials involving 23,244 participants demonstrated that GLP‐1 results in significant and clinically relevant reduction in weight, BMI and waist circumference compared to placebo [83].

Prior reviews have investigated the effect of weight loss before total joint arthroplasty (TJA), although most of these reviews only focused on one type of weight loss intervention and frequently included articles on both THAs and TKAs [47, 51, 55, 69, 75]. Two reviews [47, 69], one systematic and one rapid, were found to specifically investigate the effect of non‐surgical weight loss prior to TJA. The systematic review [69] included two randomized controlled trials and five single‐arm case series, concluding that short‐term, nonsurgical, preoperative weight loss interventions before TJA result in both statistically significant weight loss and reduced BMI prior to surgery. However, outcomes following TJA were inconsistently reported across the included studies and thus could not be analysed meaningfully. The rapid review [47] included only two retrospective studies, which had an overlapping patient population, and concluded that there was insufficient evidence to support recommending preoperative weight loss (≥5%) within the year prior to THA or TKA.

Of five reviews that were found to investigate the association of weight loss using bariatric surgery and TJA outcomes [1, 42, 51, 55, 73], two were narrative, one was systematic and two pooled the data into a meta‐analysis; furthermore, they reported conflicting findings. Murr et al. [55] reported that nine of the included clinical studies found that prior bariatric surgery decreased surgical time, length of stay, complication rates, reoperation rates and 90‐day re‐admission rates, while two of the included clinical studies found that prior bariatric surgery increased reoperation rates for stiffness and infection, and 90‐day revision rates. Furthermore, the meta‐analysis of Smith et al. [73] included five articles and concluded that there were no statistically significant differences between groups in terms of superficial and deep wound infection rates, deep vein thrombosis rates, pulmonary embolism rates, revision rates and mortality. Similarly, the meta‐analysis of Li et al. [42] included nine articles and concluded that there were no statistically significant differences between groups in terms of superficial wound infection rates, deep vein thrombosis rates, dislocation rates, periprosthetic infection rates, periprosthetic fracture rates and revision rates. Notably, there was a large overlap in included studies in these reviews, and all stated that the included articles were retrospective.

The present meta‐analysis similarly included only retrospective studies, which inherently causes inference due to potential selection bias, particularly since patients with higher BMI or comorbidity burden are more likely to be referred for bariatric surgery prior to THA. In these non‐randomized settings, referral patterns, patient motivation and institutional practices may systematically influence which patients undergo preoperative weight loss, creating groups that differ in important ways beyond weight change alone. Such baseline differences may not be fully captured or controlled for in retrospective datasets and could consequently obscure or dilute any true effect of preoperative weight loss on THA outcomes. The lack of observed differences in complication, readmission, reoperation or revision rates between weight‐loss and non–weight‐loss cohorts may partly reflect residual confounding rather than an absence of a clinically meaningful effect. Furthermore, the heterogeneity across included studies, as presented by the *I* ^2^ statistic, may limit the precision of pooled estimates, especially for outcomes that present high degrees of heterogeneity. Mid‐term complication rates, short‐term reoperation rates, short‐term revision rates and mid‐term revision rates demonstrated low heterogeneity (*I* ^2^ = 0%), suggesting relatively consistent findings across studies. Short‐term complication rates and short‐term readmission rates showed moderate heterogeneity (26% < *I* ^2^ < 50%), indicating some variability in effect estimates. In contrast, mid‐term PJI rates, short‐term SSI rates and mid‐term reoperation rates had substantial heterogeneity (51% < I^2^ < 75%). Heterogeneity may arise from a combination of patient‐, surgeon‐ and hospital‐specific factors [22, 63, 72]. Future high‐quality evidence from randomized controlled trials, or well‐designed prospective studies controlling for confounding variables, are necessary to determine whether preoperative weight loss leads to superior outcomes after THA.

It is important to highlight that nutritional factors may influence outcomes following THA, particularly among overweight and obese patients, whose weight loss not necessarily equates to improved metabolic or functional status. For instance, patients with diabetes often remain diabetic despite weight reduction [6, 79]. Similarly, individuals who undergo bariatric surgery may experience significant weight loss but may still be malnourished [10, 16]. Furthermore, GLP‐1 may contribute to muscle mass loss, potentially leading to sarcopenia [3]. These factors may contribute to the lack of clear benefit observed with preoperative weight loss in this meta‐analysis. Consequently, the use of BMI as the sole predictor of postoperative outcomes warrants re‐evaluation based on the findings of this meta‐analysis. Future studies could explore whether metabolic health parameters [40, 62, 82], such as HbA1c, CRP, the lipid profile, protein levels, as well as the risk of sarcopenia [14, 76], are more predictive of THA outcomes than weight loss alone.

The present meta‐analysis only included articles that compared outcomes following primary THA in patients who underwent preoperative weight loss compared to those who maintained the same weight. Therefore, studies that indicated that patients had undergone a weight loss intervention before THA, but did not specify if weight loss was achieved, were excluded. For example, one study reported on patients who had been prescribed GLP‐1 between 1 year and 15 days before surgery, but did not provide data confirming actual weight loss. Additionally, the timing of weight loss in relation to THA may influence outcomes. Four of the eight articles included in the present meta‐analysis did not specify the timing of weight loss. One of the included articles [72] stratified the weight loss group by the time at which weight loss occurred (<3, 3–6, 6–9, 9–12 months), and concluded that weight loss closer to THA surgery (0–9 months) can heighten risks, while losing weight a year in advance seems beneficial. The authors highlighted the importance of strategic weight management guidance for obese patients considering THA, ensuring optimal outcomes and reducing the risk of adverse events. Furthermore, a study [22] on TKA suggested that weight loss should be achieved at least nine months before surgery to decrease risks of infection. The timing of weight loss may reflect underlying metabolic stability, which could influence postoperative outcomes. If future studies confirm that earlier weight loss is associated with improved results, the development of a consensus on optimal weight loss timing prior to THA may be warranted; however, this remains speculative.

Several limitations must be acknowledged. First, all included studies were retrospective in design, limiting the strength of evidence and increasing the susceptibility to bias. Additionally, considerable heterogeneity existed across studies regarding the type of weight loss intervention, timing of weight loss relative to THA as well as the BMI thresholds used as inclusion criteria, and the magnitude of weight loss achieved. Furthermore, the follow‐up duration was not consistent across studies, and there was no standardized definition of complications. To account for the effect of follow‐up on outcomes, data were stratified by creating two follow‐up groups: short‐term (<3 months follow‐up) and mid‐term (≥24 months follow‐up). Further stratification by type of weight loss intervention was not performed, as only one article reported on bariatric surgery, two articles reported on non‐surgical interventions, one article reported on mixed interventions, and four articles did not specify the type of weight loss intervention. Moreover, although case series were initially considered in the review protocol, only comparative studies were ultimately included in the present meta‐analysis. Comparative studies provide direct head‐to‐head comparisons, allowing for more reliable assessment of differences in outcomes. Focusing on comparative studies only allowed the review to synthesize the highest‐quality available evidence, despite the observed heterogeneity among studies. While non‐comparative studies may have provided additional information, such as the effect of weight loss timing on outcomes, their inclusion would not have allowed direct comparisons between weight‐loss and non–weight‐loss groups, limiting interpretability and increasing risk of bias. Lastly, publication bias was not assessed because fewer than 10 studies were included in each meta‐analysis, thus there was insufficient data to support meaningful interpretation of funnel plots and associated tests.

## CONCLUSIONS

Preoperative weight loss in obese patients undergoing THA does not reduce the risk of postoperative complications, infections, readmissions, reoperations, or revisions compared with obese patients who did not lose weight preoperatively. These findings question routine weight loss requirements and underscore the need for individualized risk assessment over BMI alone.

## AUTHOR CONTRIBUTIONS

**Nils Meissner**: Conceptualization; investigation; funding acquisition; project administration; supervision; validation; writing—original draft; writing—review and editing. **Sonia Ramos‐Pascual**: Conceptualization; data curation; methodology; formal analysis; investigation; validation; formal analysis; writing—original draft. **Katharina Ortwig**: Investigation; writing—review and editing. **Floris van Rooij**: Conceptualization; data curation; methodology; formal analysis; investigation; validation; formal analysis; writing—original draft. **Daniel Schrednitzki**: Validation; writing—review and editing. **Johannes Stoeve**: Validation; writing—review and editing. **Andreas M. Halder**: Conceptualization; investigation; funding acquisition; supervision; validation; writing—review and editing.

## CONFLICT OF INTEREST STATEMENT

**Nils Meissner**: Research funding German Society for Orthopaedics and Trauma (DGOU), Aesculap Ag and German Innovation Fund. **Daniel Schrednitzki**: Speaker for Zimmer Biomet, Depuy. **Johannes Stoeve**: Speaker for Johnson & Johnson. **Andreas M. Halder**: Royalties from Zimmer Biomet, Depuy; Speaker for Zimmer Biomet, Depuy. The remaining authors declare no conflict of interest.

## ETHICS STATEMENT

The authors have nothing to report.

## Supporting information

supporting information.

supporting information.

## Data Availability

Data are available upon reasonable request.
