# Supplementary material for: Outcomes of total hip arthroplasty in obese patients with and without preoperative weight loss: A systematic review and meta‐analysis
Source: J Exp Orthop. 2026 Jan 21;13(1):e70651. doi: 10.1002/jeo2.70651 (PMC12821894; doi:10.1002/jeo2.70651)
Supplement: Supplementary file 1 — supporting information. [file JEO2-13-e70651-s002.pdf]

#1 hip AND (arthroplasty OR replacement)

#2 “weight loss” OR “weight reduction” OR “weight control” OR “weight change”

#3 "bariatric" OR "diet*" OR "GLP-1" OR "GLP1"

#1 AND (#2 OR #3)
