# Supplementary material for: Outcomes of total hip arthroplasty in obese patients with and without preoperative weight loss: A systematic review and meta‐analysis
Source: J Exp Orthop. 2026 Jan 21;13(1):e70651. doi: 10.1002/jeo2.70651 (PMC12821894; doi:10.1002/jeo2.70651)
Supplement: Supplementary file 2 — supporting information. [file JEO2-13-e70651-s001.pdf]

Appendix 2: Detailed funding and conflicts of interest for each study

First author, Year	Funding	COI	Description of Funding	Description of COI
Seward 2025	Yes	Yes	Grant from the Orthopaedic Research and Education Foundation (Award ID 22-053).	**Royalties:** DePuy, A Johnson & Johnson Company; Elsevier; Wolters Kluwer Health – Lippincott Williams & Wilkins; Stryker: OsteoRemedies; Springer; Zimmer Biomet **Consulting fees:** DePuy, A Johnson & Johnson Company; Stryker; Orchard Medical; Signature Orthopaedics; Enovis; Vertex Pharmaceuticals **Leadership roles:** Current Concepts in Joint Replacement (Hip Society and Knee Society); International Hip Society; Orthopaedic Research and Education Foundation (OREF); AAHKS; IOEN; Mid-America; MAOA; AAOS
Schmerler 2024	None	None	-	-
Shul 2024	NR	Yes	-	**Consulting fees:** Sage Products, Inc.; Stryker; TissueGene; 3M; Centrexion; CERAS Health; Flexion Therapeutics; Johnson & Johnson; Mirror-AR; NXSCI; Pacira; Peerwell; Pfizer-Lily; Skye Biologics; SOLVD Health; Smith & Nephew; US Medical Innovations **Research support :** National Institutes of Health **Leadership roles:** Journal of Arthroplasty; Journal of Knee Surgery; Surgical Technology International; Orthopaedics; The Knee Society; The Hip Society
LaValva 2024	Yes	Yes	Contribution of Mr. Timothy Delaney.	**Royalties, financial or material support:** Stryker; Orthodevelpment; Link; Smith and Nephew; Orthalign; Micare; Thieme; Wolters Kluwer-author **Paid employee:** from Canary Medical **Consulting fees:** Smith and Nephew; Canary Medical; Orthalign; CellSource; GK. (Tokyo, Japan) **Stocks:** in Wishbone; Naviswiss; Canary Medical; Orthalign **Research support:** Regeneron Pharmaceuticals; Pfizer **Leadership roles:** Hip Society; Knee Society; JBJS; J of ISAKOS; Journal of Joint Surgery and Research; Annals of Internal Medicine-Associate Editor
Middleton 2022	None	Yes	-	**Leadership roles:** Arthroplasty Today
Wu 2022	None	Yes	-	**Royalties:** Depuy, A Johnson and Johnson Company; Total Joint Orthopedics, INC.; Pattern Health; Restor3d **Consulting fees:** Smith and Nephew, Total Joint Orthopedics, INC., Heraeus **Stocks:** Biomech LLC **Research support:** Next Science, Zimmer **Financial support:** Lippincott Williams and Wilkins **Leadership roles:** Hip Society; American Association of Hip and Knee Surgeons; American Association of Hip and Knee Surgeons; Musculoskeletal Infection Society
Hernigou 2016	NR	None	-	-
Inacio 2014	None	None	-	-

Abbreviations: THA, total hip arthroplasty; N,
